# Lecture on Subacute Dysentery Continued—Pathology and Treatment

**Published:** 1839-12-07

**Authors:** W. W. Gerhard


### CLINICAL LECTURES.

### PHILADELPHIA HOSPITAL.

*Saturday, November* 30, 1830.

### LECTURE ON SUBACUTE DYSENTERY CONTINUED—PATHOLOGY AND TREATMENT.—PULMONARY PHTHISIS.

By W. W. Gerhard, M.D.

*No. 3—Winter Course.*

I shall bring forward to-day a case of the subacute form of dysentery, and afterwards several cases of phthisis, for the purpose of illustrating some of the various modes in which this disease commences.

The case of dysentery is one of a class not unfrequently met with during the winter months, in which not only the bowels, but the mucous membranes generally, are affected. The patient is a man of nearly fifty years of age, and an habitual drunkard. A fortnight since, he had an attack of *delirium tremens*, the symptoms of which have continued during several days after his admission into the hospital. After an exposure to cold recently, he was seized with bronchitis, and the affection of the bowels for which he is at present under treatment. The alvine discharges have been very frequent, sometimes several in the course of an hour: the pain was constant, but much more moderate than it usually is in the more acute variety of the disease. The character of the stools equally shows that it is not a case of acute dysentery: they consist of ordinary thin, fæcal matter, mixed with mucus, but at no time has either *lymph* or *blood* been present. This case is a very good exemplification of the nature of the discharges in subacute dysentery, and the changes which occur in them at different periods of the disease. At first they are either sero-mucous, or thin and feculent: subsequently their consistence and appearance become altered, and they present the characters observed at the present stage of the case before us. Blood and lymph are very seldom to be found; but in place of them, there is sometimes a grumous, fœtid matter, resembling the scrapings of a disorganized intestine, which evidently results from partial sloughing of the bowel.

Although the stools differ so strikingly from those of the acute form of the disease, subacute dysentery is still an inflammatory affection; but the degree of action is moderate, on account either of the debilitated condition of patients labouring under it, from old age, or irregular habits, or of the epidemic constitution of the season. A constitution of this sort, indeed, appears to prevail at present, imparting to epidemic diseases a tendency to assume more or less of the characters of the case which we are considering.

The principal features of the case are as follows. (Complete notes were not preserved.)

J. G., admitted November 25th. The bowels are loose; there is cough, with whitish expectoration; patient much debilitated. A dose of the oleaginous mixture was administered, and a small quantity of milk punch allowed.

26*th.*—The punch discontinued.

28*th.*—Dysenteric symptoms more severe; twelve discharges from the bowels in the course of five hours; stools thin and watery, with very little feculent matter, but very fœtid; slight pain on pressure over the abdomen; cough continues; mind confused and agitated. Six ounces of blood taken by cups placed over the colon, and the following combination prescribed:

℞. Pulv. Ipecac. et Opii gr. vj.
Hydrarg. Submuriat. gr. j.
Ext. Krameriæ gr. v. M.

To be repeated every three hours. Blisters also applied to the abdomen.

29*th.*—The Dover's powder and rhatany in the above prescription were diminished to three grains each, and the calomel to one-eighth of a grain. It was found necessary to reduce the Dover's powder, because it had produced some symptoms of narcotism; that is, contraction of the pupil, and confusion of mind, without diminishing the frequency of the stools. When such a state of things occurs, you must either discontinue opiates, or reduce the quantity administered; else the narcotic may accumulate in the system, and may cause the patient to sink suddenly and almost imperceptibly. In the early stages of acute dysentery, opium may often be given very largely without producing its characteristic effect. An attack of this variety occurred a few years since in one of the resident physicians of this hospital. He was attended by Dr. Horner and myself, and the quantity of opium was gradually increased to thirty grains a day before the least effect was produced. But in the subacute form we cannot employ such large quantities with safety, and the remedy should be withheld, or its dose diminished, as soon as it produces its specific effects. You must recollect, too, that even in the acute form of the disease, you must give it very cautiously; for mischief may in any case result from it, if administered in a careless or rash manner. The rule is, to increase it gradually, and watch carefully its effects; suspend it altogether, or diminish it greatly, the moment you find any signs of narcotism.

The Dover's powder and calomel have been the active remedies used in the treatment of this case: the extract of rhatany has been productive of no very decided benefit. The good effects of mercury in this disease, (as remarked in a previous lecture,) coincide with the occurrence of slight ptyalism,—which I find, upon examination, to have taken place in the patient before us. If it produces no amelioration of the symptoms at this characteristic period of its operation, mercury should be discontinued; and in all cases where there is sloughing of the mucous membrane, and a gangrenous fœtor of the discharges, its use should be avoided, as it then undoubtedly tends to aggravate the severity of the disease.

You must have remarked that stimuli were employed in the early treatment of this case. The patient came in, much debilitated from the effects of delirium tremens and previous dissipation, and a small quantity of milk punch was therefore allowed. In all cases of this disease, indeed, in which there is an enfeebled condition of the vital energies, it is necessary to use stimuli. This is particularly true of the malignant or sloughing variety of dysentery, between which and the subacute there is an intimate connexion. The latter has a constant tendency to pass into the former; and from what has so frequently occurred in camps and hospitals, I have no doubt that the present case would assume the malignant form, if there were many of a similar character in the wards at the same time. If such a change should supervene, it would be necessary, as I have already remarked, to discontinue the use of mercurials, and to rely principally upon Dover's powder and stimuli.

The cupping in this case produced only moderate benefit, whereas the blister operated very advantageously. It is one of those instances of moderate, but obstinate inflammatory action, to which blisters are so peculiarly adapted. Cupping or leeching would be better applicable to the more violent and acute cases of the disease.

The woman who was brought before you two weeks since as a case of acute dysentery, and was treated with mercurials, is now quite well. The man who was labouring under the subacute form of the disease, at first improved considerably, by the use of Dover's powder and astringents, but afterwards sank again, and died on the 28th inst. The results of the post mortem examination are as follows, and show the cause of death:

I now present to you the lesions met with in the intestinal canal.

The mucous coat of the rectum is of a bluish colour, and softened. As we pass up the colon, we find extensive ulcerations, some of which are cicatrized. There is a large cicatrix near the sigmoid flexures; the newly formed membrane is thin and bluish; the old membrane, on the contrary, is much thickened, and to a still greater degree are the cellular and muscular coats. Farther up the ulcers are scattered about, of small size; the mucous membrane softened. Near the cœcum the morbid changes are of more recent date: the mucous membrane is highly injected, and patches of lymph are here and there observed. The ulcers are in the acute stage of their progress; they are of a rounded form, and are seen to have commenced in the follicles of the intestine. We have here exemplified the different appearances in the acute and chronic forms of dysentery: in the upper part of the colon, where the former condition prevailed, the mucous membrane is of a bright red colour, as in acute inflammation of this tissue generally; but in the lower part, where the disease had become chronic, the colon is bluish. This examination also illustrates a remark which I made in a former lecture, that dysentery usually commences in the lower portion of the colon, and proceeds upwards in its course.

The disease has also passed into the ileum, which is in a state of acute inflammation. There are bright red spots, and patches of lymph, scattered over the mucous membrane, for some distance from the ileo-cœcal valve. The glands of Peyer are altogether or nearly intact; in which circumstance you perceive a striking difference from the inflammation of the ileum which occurs in typhoid fever. This acute inflammation of the small intestine, supervening in the course of the dysentery, was the immediate cause of death. The mucous membrane becomes gradually more healthy as we ascend, but is more or less softened.

The stomach also presents marks of inflammation. At the splenic extremity the coats are very thin, and much softened; this condition was in a great measure produced by the action of the gastric juice, which had become altered in constitution, and excessively acid; the membranes at this point have an acid smell, and produce an unusually powerful reaction upon test paper. Throughout the remaining portions of this viscus the coats are white and opaque, from an alteration both in the fluids and the structure. There are dark red patches in several places. Near the pylorus there is a puckering of the mucous membrane, produced by a cicatrix. This membrane is generally softened, but the colour is not particularly reddened. The coats of the pylorus are thickened and indurated, and there is a scirrhous formation in the cellular tissue; its fibrous character is, however, not yet distinctly marked. The symptoms produced by scirrhus of the pylorus, are difficulty of digestion, and vomiting from time to time, which may be so severe and repeated as to produce death. It may be remarked that these scirrhous affections are frequently developed in constitutions where the diathesis has been latent, by depression of mind, particularly in old persons. This is illustrated by the instance of Napoleon; and I recollect an equally striking case occurring in a Swiss emigrant.

The liver is also affected in the present case, but lesions of this organ accompany dysentery far more frequently in hot than in cold and temperate climates. Its colour is a pale yellow, and it appears to be in the first stage of that alteration called the *fatty degeneracy*. The tests of this condition are the greasy appearance of the scalpel when drawn through the substance of the liver, and the bright flame produced by burning a piece of paper which has been moistened with the fatty fluid. The acini are also observed to be very distinct, and surrounded by vessels, producing an appearance of great vascularity and incipient inflammation of the liver. The fatty degeneracy in this case was probably produced by the habitual intemperance of the patient, and subsequently increased by the dysentery.

The spleen, which is so often found greatly enlarged and softened in various diseases, is here nearly of the natural size, but the texture is softened. The cause of this change in the spleen is not well understood, but it appears to be in some way connected with an alteration of the blood. The spleen probably performs an important part in the formation of this fluid; but what the precise office is, cannot be yet ascertained.

From this examination, you will perceive how readily the most extensive ulcerations of the mucous membrane of the intestine will cicatrize, provided the muscular coat is not exposed by them.

### PULMONARY PHTHISIS.

I next proceed to bring to your notice several cases of phthisis pulmonalis, with the view of exemplifying some of its modes of origin;—this is the more appropriate, as the present season gives rise to few acute diseases.

*Case 1st.*—You may recollect that this man came before you on the 23d inst., with many symptoms resembling those of intermittent fever. Since that time the skin has continued hot, with occasional sweats; the pulse has usually beaten one hundred and sixteen in the minute; yesterday, ninety-six; it is also quick and irritable, jerking, and easily felt, but small, and not corded. This character of the pulse is often important as a diagnostic sign: it occurs in chronic diseases, or in acute diseases gradually passing into a chronic state, accompanied by general disorder of the system. It is most frequently observed in incipient phthisis, particularly when this disease is attended by pleurisy. The respiration is also much more hurried than natural, being performed from thirty to thirty-six times a minute. Cough frequent; expectoration thin, white, and small in quantity; tongue at present somewhat pale, and gradually cleaning. The respiration is resuming its natural character in the lower part of the right lung, owing to the decline of the pleurisy; but it remains rude in the upper lobe of the lung; a sign corresponding to the tuberculous condition of the part. The action of the heart continues exaggerated, and the second sound is still absent. The difficulty of walking also continues, but in a somewhat less degree; it is dependent on an acute affection of the spine.

The treatment of this case has consisted in the application of a blister to the cardiac region, and the administration of digitalis and Dover's powder, in the proportion of one-third of a grain of the former to three grains of the latter, the dose being repeated four times a day. Considerable advantage has resulted from these measures; the inflammation which had existed being in a great measure overcome, and the inordinate action of the heart diminished. The digitalis and Dover's powder were given particularly with a view to the latter effect, and to allay the irritability of the pulse. This combination sometimes produces a dryness of the mouth, and of the alimentary canal generally, which is unfavourable to the due performance of the digestive functions: but it is by far the best mode of administering these remedies.

I have introduced this case as an example of tubercular disease commencing with pleurisy. The tubercular deposition seems to be a result of the inflammation, as truly as are its ordinary consequences, effusion, adhesion, &c. In most patients the latter conditions would be the only effects of the inflammation; but in the present case there existed a cachectic condition of the system, predisposing it to the occurrence of phthisis: hence this disease was developed as a consequence of the inflammation. The inflammation which thus produces phthisis, may be seated in any of the tissues of the lungs: thus, it may be a bronchitis, or a pneumonia, as well as a pleurisy. But it usually affects a *serous* membrane: therefore, pleurisy is more frequent than the other pulmonary inflammations preceding phthisis, just as peritonitis also is often the primary lesion in tuberculous deposits in the peritoneum. But these cases of inflammatory phthisis are by no means so frequent as those which commence gradually and slowly. Of this more common form we have an example in the next case.

*Case 2d.*—The patient, who is a printer, has for a long time had a slight cough, and other symptoms of incipient phthisis. During the last summer, there was a great aggravation of all the symptoms, accompanied by a slight inflammation of the bronchial tubes; and the patient is now scarcely able to leave his bed. In this case, it is evident that the inflammation was the consequence, not the cause of the phthisis, as in the preceding case. With regard to those cases of phthisis which I suppose to be caused by inflammation; it may be argued by those opposed to this view of the origin of the disease, that the phthisis *existed*, but in a *latent* form, previously to the commencement of the inflammation. But those cases are exceedingly rare, in which the disease does not declare itself, either by some symptoms, or by physical signs. Hence, I feel compelled to believe, in contradiction to my former opinion, that phthisis may arise from inflammation, occurring in constitutions predisposed to the formation of tubercles.

*Case 3d.*—This woman has had a slight cough for four years; about three months since, after exposure to wet and cold, the cough and other symptoms were greatly aggravated. These circumstances are sufficient to show that, in this case also, the inflammatory symptoms supervened in the course of the phthisis, instead of preceding it.

*Case 4th.*—This woman also has had a cough of four years standing, which became very bad during the summer; after working in an exposed situation. During the last week there has been flying pains under the ribs of the right side. These pains arise from the slight and shifting pleurisy so often met with in cases of tubercular disease. These pleurisies are generally dry; that is, unattended by effusion. The expectoration is copious: the sputa are in round masses, and of the kind called, from this circumstance, nummular; it usually sinks in water, but sometimes floats, from its containing particles of air; it consists of pus, mixed with mucus, and sometimes a portion of broken-down tubercular matter.

In both these women, the skin has assumed a dusky, earthy colour, entirely distinct from the paleness which accompanies it; this hue is almost peculiar to phthisis. The sclerotica has that light blue tinge which is so frequently met with in certain cases of phthisis. The appetite has remained good: indeed a good appetite, persisting for a long time in a patient affected with cough and gradually becoming thin and pale, is one of the most constant indications of commencing phthisis pulmonalis; in many cases the appetite is morbidly great. One of the patients is *jaundiced.* This probably arises from a fatty degeneracy of the liver, accompanied by enlargement; a condition very frequently occurring in this disease; more particularly in female patients. The jaundice arising from this cause must be carefully distinguished by the practitioner from that which depends upon primary affections of the liver, and is totally unconnected with any disease of the lungs. I have seen many cases of phthisis in which the liver was supposed to be the sole cause of the mischief. It is true, that in a large proportion of these patients the liver was fatty, but this condition arises from the phthisis, and is neither a primary lesion, nor one in itself of much importance.

*Case 5th.*—This man has been very recently admitted into the hospital. He is much emaciated, and has had cough for four weeks: the disease is therefore probably phthisis. This probability is rendered almost certain by the respiration being very deficient under one clavicle, and feeble under the other also. But there are as yet no signs of the existence of a cavity. The patient's account of the manner in which the disease commenced is this: After having been engaged at hard labour, out of doors, during the day, he was awakened from sleep in the night by a sudden fit of coughing and hæmoptysis; in a short time he discharged in this way a pint of blood. The hæmoptysis lasted several days; but for the last three weeks the sputa have contained no blood, but only a frothy matter. This is an example of the *hæmorrhagic* variety of phthisis. The hæmorrhage with which the disease commenced arose from the mucous membrane of the bronchial tubes. In such cases the hardening of the pulmonary tissue, from the deposition of tubercular matter, does not generally commence early in the progress of the disease; or rather, it does not soon proceed to a high degree of induration in any particular portion of the lung.

The patient is a shoemaker: and we may remark that shoemakers and printers are particularly subject to phthisis, from the confined and sedentary nature of their occupations. His constitution has always been delicate; but he has never before had cough or pain in the chest, except after occasional exposure to cold. Immediately previous to the present attack, he was in the enjoyment of his usual degree of health.

*Case 6th.*—The patient is, like the last, a shoemaker by trade, aged 42. He has had cough for nearly two years, but it has been generally unattended with pain; there has been merely a sense of oppression at the lower end of the sternum. In February, 1838, he had an attack of pleurisy, which has left behind it the usual sign of contraction of the chest. He had no cough previously to the attack of pleurisy, except for a short time, about six months before, and occasionally during spells of intermittent fever. The case, therefore, appears to be one of the inflammatory variety of phthisis, having been preceded, as in the first of these cases, by inflammation of the pleura.

*Case 7th.*—This is another example of phthisis commencing suddenly, with an attack of inflammation. The patient, a man 41 years of age, has had cough for eighteen months; it began after he had been working at the engines at a fire; had previously been a stout, healthy man, and had never had a cough of any duration. The attack was one of considerable severity, but the patient was not confined to bed by it; he did light work for a year afterwards, but for the last six months has been unable to perform any kind of labour. He is much emaciated, and presents all the signs of confirmed phthisis.

The cases of phthisis arising from inflammation are much more frequently found in men accustomed to hard labour than in others, and especially in those past the age of five and thirty. In younger persons, especially females, the disease is more frequent, and more commonly begins in a slow and insidious manner; inflammation may, and often does, occur in these cases, but then it is strictly secondary, and depends either upon the irritation of tubercles in the lungs, or the accompanying fever.

